# Neurofunctional Correlates of Environmental Cognition: An fMRI Study with Images from Episodic Memory

**DOI:** 10.1371/journal.pone.0122470

**Published:** 2015-04-14

**Authors:** Aline Vedder, Lukasz Smigielski, Evgeny Gutyrchik, Yan Bao, Janusch Blautzik, Ernst Pöppel, Yuliya Zaytseva, Edmund Russell

**Affiliations:** 1 Human Science Center, Ludwig-Maximilians-Universität (LMU), Munich, Germany; 2 Institute of Sociology, (LMU), Munich, Germany; 3 Institute of Medical Psychology, (LMU), Munich, Germany; 4 Parmenides Center for Art and Science, Pullach, Germany; 5 Institute of Clinical Radiology, (LMU), Munich, Germany; 6 Department of Psychology, Peking University, Beijing, People’s Republic of China; 7 Key Laboratory of Machine Perception, Peking University, Beijing, People’s Republic of China; 8 Moscow Research Institute of Psychiatry, Moscow, Russia; 9 Department of History, University of Kansas, Lawrence, United States of America; Hospital General Dr. Manuel Gea González, MEXICO

## Abstract

This study capitalizes on individual episodic memories to investigate the question, how dif-ferent environments affect us on a neural level. Instead of using predefined environmental stimuli, this study relied on individual representations of beauty and pleasure. Drawing upon episodic memories we conducted two experiments. Healthy subjects imagined pleasant and non-pleasant environments, as well as beautiful and non-beautiful environments while neural activity was measured by using functional Magnetic Resonance Imaging. Although subjects found the different conditions equally simple to visualize, our results revealed more distribut-ed brain activations for non-pleasant and non-beautiful environments than for pleasant and beautiful environments. The additional regions activated in non-pleasant (left lateral prefrontal cortex) and non-beautiful environments (supplementary motor area, anterior cortical midline structures) are involved in self-regulation and top-down cognitive control. Taken together, the results show that perceptual experiences and emotional evaluations of environments within a positive and a negative frame of reference are based on distinct patterns of neural activity. We interpret the data in terms of a different cognitive and processing load placed by exposure to different environments. The results hint at the efficiency of subject-generated representations as stimulus material.

## Introduction

How do different environments affect us on a neural level? This study capitalizes on individually unique mental imagery to address the issue of environment in a novel way. Instead of using predefined stimuli, this study focuses on the individual and his/her conceptualization of beautiful and pleasant environments. This new approach considers the complexity and inter-individual variability of the relationship between human beings and their environment. In addition, by collecting neural data we scrutinize the indirect effects of different environmental conditions.

Previous empirical research on the relationship between humans and their environment has mostly focused on direct effects and physical risks (e.g., pathogens and toxic substances), as well as on explicit judgments of environmental criteria. But a growing body of evidence has shown that environments have significant indirect effects too [1], [2], [3]. For instance, interacting with nature brings faster recuperation following stress reactions than interacting with urban environments, as shown in self-reports, as well as by tracking appropriate physiological reactions, such as systolic and diastolic blood pressure (SBP, DBP), pulse transit time (PTT), spontaneous skin conductance response (SCR), frontalis muscle tension (EMG), and heart-period data over time [4], [5], [6].

The aesthetic appeal of an environment and its effect upon individuals has been studied and incorporated into various experimental projects in healthcare facilities [7]. It has been documented that figurative art has an impact on measurable health indicators. Patients recovering from open-heart surgery who lived in rooms with images depicting nature, in comparison to those who lived in rooms with blank walls or decorated with abstract paintings, experienced less anxiety and consumed less pain medication [8]. Even differences between artistic and non-artistic images [9] or distinct artistic styles [10] can be linked to different processing mechanisms.

Natural environments may provide high levels of inherently fascinating stimuli which engage involuntary attention, thus giving the directed attention fatigue an opportunity to rest and recuperate [11]. In line with this, environmental psychologists have suggested that an innate love of nature, biophilia, makes nature essential for human well-being [12]. The biophilia hypothesis treats an environmental feature (nature) as an independent variable to which people respond in a universal way.

Therefore, to investigate the impact of environmental conditions on affect and cognition, it is necessary to focus on implicit psychological processes in response to sensory stimulation. Probably the most salient response to an environmental condition is stress. Non-effective stress-combatting strategies may affect our health through chronic arousal, suppression of the immune system, or other forms of allostatic load [13], [14]. In line with this, the concept of effortless processing (e.g. [15]) addresses the cognitive and neural demands that environments place on individuals. It can be assumed that health-promoting environments require less cognitive and neural effort to process than environments that are detrimental to health. Accordingly, the brain of an individual in a health-promoting environment might show less physiological activity than the brain of the same individual in a health-detrimental environment. This degree of physiological activity can be measured indirectly with functional magnetic resonance images of blood-oxygen-level-dependent (BOLD) signals. Using fMRI, Lederbogen et al. (2011) [16] showed that urban upbringing, as well as living in a city, has dissociable impacts on social evaluative stress processing in humans. The authors associate current city living with increased amygdala activity [16]. According to their study, urban upbringing affects the perigenual anterior cingulate cortex, a key region for the regulation of amygdala activity, negative affect, and stress [16]. The study reveals a strong connection between environmental settings and neural mechanisms.

Accordingly, previous research provides a coherent picture of the strong interconnectedness between environmental conditions and well-being. Nevertheless, experimental evidence is required to explain the neural mechanisms that could be responsible for the distinct effects of different environments [17]. Cognitive neuroscience can also provide insight into implicit perceptual experiences of aesthetics, its neural foundation, and its relation to other cognitive processes [18], [19].

Previous experimental studies typically asked subjects to respond to environmental stimuli preselected by experimenters. These studies often tested for universal differences, while masking variation among individuals. Albeit external stimuli, such as pictures, are highly important in the evaluation of environmental cognition processes and anthropological universals, these stimuli do not touch upon the aspects that might be characteristic and unique to individual subjective representations of positive and negative environments. There exist cultural and individual differences in regard to attractive and natural elements of environmental surroundings [20]. Even personality traits may modulate aesthetic experiences [21].

Consequently, this study aims to replace universal conceptualizations of beauty and pleasure with individual, subject-generated definitions of beautiful/non-beautiful and pleasant/non-pleasant environments to examine the neurocognitive processing of positive and negative environments. We asked subjects to tap into their rich reservoirs of episodic memory through mental imagery, an ability to generate mental representations of a stimulus in the absence of the stimulus itself, but preserving its perceptible properties. A rich remembered experience is a defining feature of episodic memory recall [22]. Episodic memory capitalizes on the interlinking of implicit and explicit knowledge frames [19]. Accumulated findings document that imagery and perception draw on most of the same neural machinery [23], including both cognitive level [24], [25], [26] and neural topography [27], [28], [29]. Belardinelli and her colleagues (2009) [30] demonstrated that the ability to generate vivid mental images influences both the format and the neural activation levels of image formation. Highly-vivid subjects, in comparison to lowly-vivid subjects create even more analogic images, characterized by higher levels of neuronal equivalence with perception.

Capitalizing on the methodological power of functional magnetic resonance imaging, controlled introspection in the form of episodic memories can be used to access the individual environmental experiences of the study participants. Accordingly, in our study, we were interested in the impact of different environments on cognitive processes. Which physiologic mechanisms can be identified for the positive or negative effects of environments? With the decision to investigate beautiful/non-beautiful as well as pleasant/non-pleasant environments, the study aim was to explore whether certain neural patterns are typical for positive and negative frames of reference. Both pleasure and beauty refer to the appeal of an environment on the perceiving person. One condition was chosen that describes the external sensation linked to the experience of an environment (beautiful/non-beautiful) while the other condition describes the internal sensation (pleasant/non-pleasant) linked to the experience of an environment. Considering previous research, we hypothesized that positive and potentially health-promoting environments require different mental effort to process than negative environments.

## Methods

### Participants

From a pool of 150 volunteers, we selected 16 right-handed (as assessed by the Edinburgh Handedness Inventory [31]) subjects (nine females, mean age 23.1), fluent in German language, who had scored high on the ability to generate vivid mental representations as assessed in the Questionnaire Upon Mental Imagery ([32], mean score 54.4). The questionnaire assesses the vividness of inner representations in seven sensory modalities (visual, auditory, cutaneous, kinaesthetic, gustatory, olfactory and organic). The short version we used for the current study, has 35 items [32], five for each of seven modalities. The responses are rated on a seven point scale where (1) = perfectly clear and as vivid as the actual experience, and (7) = no imagery present. The study participants were informed about the standard fMRI exclusion criteria (implanted metal objects in the bodies, history of neurological or psychiatric disorders, pregnancy, claustrophobia), and a written consent was obtained from all participants. The Ethics Committee of the Medical Faculty at the University of Munich approved the protocol. The study was conducted in accordance with the Declaration of Helsinki.

### Stimuli

Prior to the fMRI experiment, all 16 participants took part in a one-hour preparatory training session in which they were instructed first to imagine with their eyes closed and then to describe in writing self-chosen beautiful (German term: schön) and non-beautiful as well as pleasant (German term: wohltuend) and non-pleasant environments. Subjects were free to choose scenes from their own individual experience. Written recordings were encouraged to help them refresh and store images and sensations in memory for further use in the main experiment. Inside the scanner, participants viewed the simplified instructions via a mirror attached to the head-coil on a LCD screen.

### Procedure

We performed fMRI scans while the subjects imagined four sceneries in four experimental conditions (beautiful and non-beautiful; pleasant and non-pleasant). The first experiment required the participants to imagine beautiful and non-beautiful environments while the second experiment required the imagination of pleasant and non-pleasant environments. Half of the participants had to imagine beautiful and non-beautiful environments first while the other half had to imagine pleasant and non-pleasant environments first. A block design was used with 8 blocks per each condition, each block comprising instructions for the subjects.

Tasks and procedures were discussed in detail in the training sessions before the experiment. The order of blocks was pseudo-randomized (Presentation, Neurobehavioral Systems, USA). The instructions were presented for 2 seconds, followed by a black screen displayed for 18 seconds while subjects imagined the different environmental conditions. Since the subjects were asked to keep their eyes closed during each imagination period, an acoustic signal marked the end of the visualization session. Next, the participants had 2 seconds to respond to a control question asking whether the imagined scenery was easy or difficult to imagine by pressing either “yes” or “no” on a button (LUMItouch, Photon Control Inc, Canada). With this control question we could assesses the intensity of the task, obtain an immediate and intuitive answer, and encourage the concentration and attention of the study participants in the scanner. A fixation asterisk appeared on the screen for 6 seconds after each block.

### Scanning and data analysis

The experiment was conducted with a 3T system (Philips ACHIEVA, Best, The Netherlands) at the University Hospital LMU Munich. Foam cushions securely, but comfortably, fastened the subject’s head to minimize movements. As anatomical reference and to detect potential morphological anomalies, a T1-weighted, magnetization-prepared rapid gradient echo (MPRAGE) sequence was performed: repetition time (TR) = 2400 ms, echo time (TE) = 3.06 ms, flip angle (FA) = 9°, number of slices = 160, matrix = 224 x 256, spatial resolution 1 x 1 mm. Structural images were acquired in sagittal orientation. For BOLD imaging, a T2*-weighted EPI sequence was used (TR = 2000 ms, TE = 35 ms, FA = 90°, 28 axial slices covering whole cerebrum, slice thickness = 4 mm, inter-slice gap = 0.4 mm, ascending interleaved acquisition, FOV = 230 x 230 mm, matrix = 128 x 128, in-plane resolution = 1.8 x 1.8 mm). In total, 248 functional volumes were acquired. Functional images were acquired in axial orientation (parallel to the anterior commissure—posterior commissure [AC-PC] line).

To account for T1 saturation effects, the first five volumes in each run were excluded from further analysis. The functional images were realigned, co-registered, and spatially normalized into standard stereotaxic space (EPI template; Montreal Neurologic Institute, MNI), re-sliced to 2 x 2 x 2 mm voxels, and smoothed with an 8 mm full-width at half maximum (FWHM) Gaussian kernel using SPM8 software (Statistical Parametric Mapping; http://www.fil.ion.ucl.ac.uk/spm). T-contrast images were created versus baseline for each subject at the first level. After estimation of the random-effects second level (full-factorial design), statistical parametric maps were thresholded at p <.001 (corrected for multiple comparisons at cluster-level using the family-wise error correction at p (FWE) <.05).

The clusters were anatomically described using the AAL atlas (Automated Anatomical Labeling of Activations [33]; AAL Toolbox for SPM8, http://www.gin.cnrs.fr/spip.php?article217).

## Results

### Experiment 1: Beautiful and non-beautiful environments

Subjects reported that both environmental conditions were easy to imagine (*M* = 84.9, SD = 13.7% for the beautiful and *M* = 79.5, SD = 14.7% for the non-beautiful setting). The difference was not statistically significant (*t*(7) = 0.77, *p* >.05).

Compared to baseline, conjunction analysis revealed activations in the supplementary motor area as well as in the superior frontal gyrus for both beautiful and non-beautiful conditions.

For the non-beautiful condition as compared to the beautiful condition, activations were registered in the anterior cortical midline structures (anterior cingulate, medial prefrontal cortex, supplementary motor area), right superior frontal gyrus, as well as in the left superior, middle, inferior frontal gyri, insular cortex, orbitofrontal cortex and temporal pole. The opposite comparison, beautiful vs. non-beautiful condition, revealed no significant activations.

**Fig 1:**
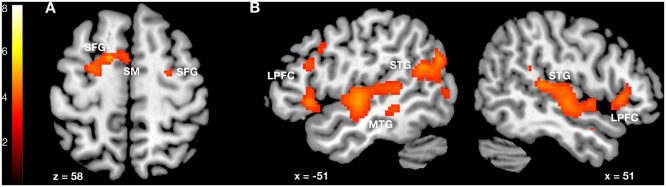
Neurofunctional processing of beautiful and non-beautiful environments. (A) Conjunction of beautiful and non-beautiful environments versus baseline and (B) higher neural activation for non-beautiful environments. SM = supplementary motor area, SFG = superior frontal gyrus, LPFC = lateral prefrontal cortex, STG = superior temporal gyrus, MTG = middle temporal gyrus.

Fig 1 and Table 1 contain detailed quantitative information about the observed relationship.

**Table 1:** Neurofunctional processing of beautiful and non-beautiful environments.

			Coordinates			
Brain region	cluster	kE	x	y	z	z-value
*conjunction of (beautiful > baseline) and (non-beautiful > baseline)*						
	1	239				
L supplemental motor area			-14	4	60	4.40
L superior frontal g.						
*non-beautiful > beautiful*						
	1	856				
R insular cortex			42	24	-8	5.34
R inferior frontal g.						
R middle frontal g.						
	2	4301				
L superior temporal g.			-34	14	-20	4.75
L insular cortex						
L inferior frontal g.						
L. middle temporal g.						
L. angular g.						
	3	1859				
Precuneus			4	-52	54	4.69
Cuneus						
Middle cingulate cortex						
	4	1796				
R superior temporal g.			56	-10	-6	4.59
R thamalus						
	5	7580				
Anterior cingulate cortex			8	40	24	4.55
L supplementary motor area						
L superior frontal g. (medial part)						
R superior frontal g. (medial part)						
L middle frontal g.						
	6	374				
R hippocampus			36	-8	-18	4.38
R amygdala						
	7	506				
L precentral g.			-42	4	48	4.06
L inferior frontal g.						
L middle frontal g.						
	8	554				
R angular g.			38	-72	48	3.85
R superior parietal g.						

*Note*. kE = size in voxels (2 x 2 x 2 mm). R = right, L = left, g. = gyrus. The *x*, *y* and *z* coordinates are in the MNI stereotactic space. *p* <.001.

### Experiment 2: Pleasant and non-pleasant environments

Subjects reported that both environmental conditions were easy to imagine (*M* = 95.3, SD = 6.2% for the pleasant and *M* = 93.0, SD = 12.9% for the non-pleasant setting). The difference was not statistically significant (*t*(7) = 0.72, *p* >.05).

Compared to baseline, conjunction analysis revealed activations in the visual and sensorimotor cortex over baseline for both pleasant and non-pleasant conditions.

Functional magnetic resonance imaging showed significantly more cortical activations when subjects imagined non-pleasant environments than when they imagined pleasant environments. The higher blood-oxygen-level-dependent (BOLD) signal appeared primarily in the left prefrontal cortex (the ventrolateral part including Broca’s speech area). The opposite comparison, pleasant vs. non-pleasant condition, revealed no significant activations.

**Fig 2:**
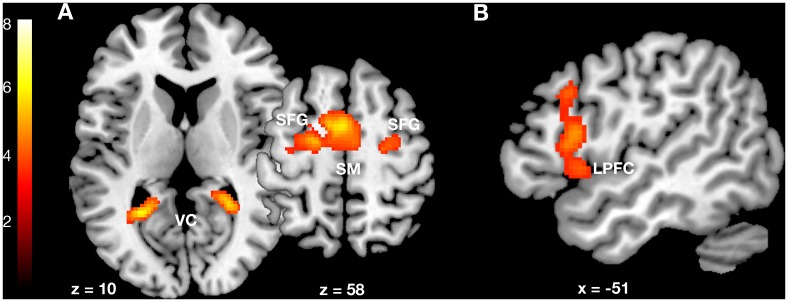
Neurofunctional processing of pleasant and non-pleasant environments. (A) Conjunction of pleasant and non-pleasant environments versus baseline and (B) higher neural activation for non-pleasant environments. VC = visual cortex, SM = supplementary motor area, SFG = superior frontal gyrus, LPFC = lateral prefrontal cortex.

Fig 2 and Table 2 contain detailed quantitative information about the observed relationship.

**Table 2:** Neurofunctional processing of pleasant and non-pleasant environments.

			Coordinates			
Brain region	cluster	kE	*x*	*y*	*z*	*z*-value
*conjunction of (pleasant > baseline) and (non-pleasant > baseline)*						
	1	358				
L precuneus			-28	-54	14	5.89
L calcarine						
	2	480				
R precuneus						
R calcarine			20	-44	16	4.95
	3	1045				
L supplementary motor area			-32	-14	60	3.30
R supplementary motor area						
L superior frontal g.						
R superior frontal g.						
L precentral g.						
R precentral g.						
R parahippocampal						
*non-pleasant > pleasant*						
	1	810				
L precentral g.			-36	4	62	3.18
L middle frontal g.						
L inferior frontal g.						
L. superior temporal g.						

*Note*. kE = size in voxels (2 x 2 x 2 mm). R = right, L = left, g. = gyrus. The *x*, *y* and *z* coordinates are in the MNI stereotactic space. *p* <.001.

## Discussion

The results of this study show that a positive and a negative frame of reference elicit distinct neural patterns of environmental cognition. We assume that non-beautiful and non-pleasant environments demand more mental processing than beautiful and pleasant environments. Although behavioral data illustrates that subjects found the different environmental conditions equally easy to visualize, imagining non-beautiful and non-pleasant environments activated more brain areas, revealing a greater diversification of brain reactivity than beautiful and pleasant environments. The results correlate with previous propositions to explain the experience of negative environments as characterized by the demand on more mental resources than the experience of positive environments. Taken together, cognitive experiences within different environmental frames are based on different patterns of neural activity.

The perception of non-pleasant environments is characterized by a higher neural response in the left prefrontal cortex. Previous research has identified the left prefrontal cortex as a key structure in the executive-control system [34]. The hypothesis that cognitive costs correlate with activation of brain areas responsible for executive control, particularly the left prefrontal cortex, has found solid support in functional MRI experiments [35]. Thus, the weaker BOLD signal for pleasant mental scenarios in the left lateral prefrontal cortex suggests a lower cognitive load, which indicates that positive and potentially health-promoting environments might require less effort in neural processing.

A similar pattern of increased neural activity can be observed for the processing of non-beautiful environments. The non-beautiful condition elicits activity in the orbitofrontal cortex (OFC) that can be linked to the online monitoring of behavior based on emotional cues and somatic signals [36]. Other areas activated in the non-beautiful condition indicate top-down cognitive control (right superior frontal gyrus and left inferior frontal gyrus) [37], [38] and response to an increase in executive demand in the working memory system (left superior frontal gyrus) [39]. In addition, a stronger activation of the supplementary motor area (SMA) can be observed. The SMA has been suggested to be a motor-limbic interface contributing to the transformation of visually-triggered emotional experiences into motor actions [40]. The non-beautiful condition also causes increased activity in the anterior cortical midline structures, including the anterior cingulate cortex (ACC) and the medial prefrontal cortex (mPFC). The role of the ACC-mPFC network (having vast projections to the amygdala) in the appraisal of negatively charged emotional stimuli has been suggested previously [41]. Many studies highlight that the same circuitry is also involved in emotion regulation [42] [43]. Activity in the ACC has also been linked to a conscious mental effort related to cognitive processing [44]. Thus, an enhanced signal in the ACC and ACC-mPFC network may imply that the processing of non-beautiful settings demands increases in top-down cognitive control and regulatory mechanisms. In line with this, the stronger BOLD signal in the left insular cortex for the processing of non-beautiful environments can be attributed to higher emotional arousal. There is evidence that the insula plays an important role in generating anticipatory signals, which are critical for learning about aversive outcomes [45]. Activity in the left insular cortex may point to the mobilization of greater neural resources toward discomfort-inducing, unappealing, and potentially aversive events.

Considering the data, we propose that interacting with a negative environment requires an additional investment in emotion processing, cognitive control, and motor function. The neural results locate causal mechanisms for the fundamental effects of different environments and support the effortless processing concept of cognition [15]. This argumentation finds additional support from the results of the study conducted by Martinez-Soto and colleagues [46] on the neural correlates of restorative environments. The exposure to low restorative pictures revealed a more distributed brain pattern (same as in our study) and activated the cortical areas related to direct, endogenous and top-down attention, in comparison with the high restorative pictures activating more involuntary, exogenous and bottom-up attentional resources.

The implications of our study can be considered with respect to the discussion on the impact of the physical environment on cognitive processing, evidence-driven studies of human-environment interaction, and even health-related parameters.

The task to imagine beautiful/non-beautiful and pleasant/non-pleasant environments can be considered to be a complex cognitive process. Thus, it is plausible that no single cortical region can wholly account for the different experimental conditions. To our knowledge, this is the first study to measure the influence of mental representations of different environments on a neural level using functional MRI.

Some limits of the study point to areas for future research. Our focus was an inner experience based on a conscious, quasi-perceptual phenomenon of mental imagery fed by episodic memory. Extending the data to real perceptual experiences should be performed with caution in spite of the fact that similar neural structures are involved in both instances. The results also raise questions about temporal frames and the long-term neural effects of different environmental conditions [47]. Furthermore, intercultural comparisons would help to clarify the extent to which certain effects of environmental cognition can be generalized. A formulation of evidence-based postulates on how to mold and organize physical space for the benefit of individuals should be the aim of future research. Accordingly, the most urgent challenge for future research will be to elucidate the neural mechanisms through which environments affect our well-being and health.

The results of this study highlight the difference in neural activity linked to the experience of positive and negative environments. The outcome is remarkable, considering that subjects were simply imagining different environments rather than responding to real environments. Taken together, the observed patterns of brain responsiveness have led us to conclude that the neurofunctional processing of positive environments in comparison to negative environments might involve less processing costs, less cognitive monitoring, and emotion regulation.

## Supporting Information

S1 Graphic Schematic representation of the experimental design for one fMRI session. (JPG)
