# Supplementary figures and images for: Neurofunctional Correlates of Environmental Cognition: An fMRI Study with Images from Episodic Memory
Source: PLoS One. 2015 Apr 14;10(4):e0122470. doi: 10.1371/journal.pone.0122470 (PMC4397013; doi:10.1371/journal.pone.0122470)

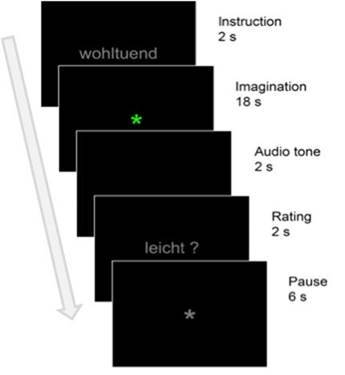

Supplement: S1 Graphic — (JPG) [file pone.0122470.s001.jpg]
